# Abnormal loading and functional deficits are present in both limbs before and after unilateral knee arthroplasty

**DOI:** 10.1016/j.gaitpost.2017.04.008

**Published:** 2017-06

**Authors:** A.J. Metcalfe, C.J. Stewart, N.J. Postans, P.R. Biggs, G.M. Whatling, C.A. Holt, A.P. Roberts

**Affiliations:** a The Robert Jones and Agnes Hunt Orthopaedic Hospital, Oswestry, United Kingdom; b The Arthritis Research UK Biomechanics and Bioengineering Centre, Cardiff School of Engineering, Cardiff University, United Kingdom; c Warwick Medical School, The University of Warwick, United Kingdom; d The Institute for Science and Technology in Medicine, Keele University, United Kingdom

**Keywords:** Knee osteoarthritis, Knee replacement, Gait analysis, Cardiff classifier, Bilateral

## Abstract

Unilateral knee replacement is often followed by a contralateral replacement in time and the biomechanics of the other knee before and after knee replacement remains poorly understood. The aim of this paper is to distinguish the features of arthritic gait in the affected and unaffected legs relative to a normal population and to assess the objective recovery of gait function post-operatively, with the aim of defining patients at risk of poor post-operative function. Twenty patients with severe knee OA but no pain or deformity in any other lower limb joint were compared to twenty healthy subjects of the same age. Gait analysis was performed and quadriceps and hamstrings co-contraction was measured. Fifteen subjects returned 1 year following knee arthroplasty. Moments and impulses were calculated, principal component analysis was used to analyse the waveforms and a classification technique (the Cardiff Classifier) was used to select the most discriminant data and define functional performance. Comparing pre-operative function to healthy function, classification accuracies for the affected and unaffected knees were 95% and 92.5% respectively. Post-operatively, the affected limb returned to the normal half of the classifier in 8 patients, and 7 of those patients returned to normal function in the unaffected limb. Recovery of normal gait could be correctly predicted 13 out of 15 times at the affected knee, and 12 out of 15 times at the unaffected knee based on pre-operative gait function. Focused rehabilitation prior to surgery may be beneficial to optimise outcomes and protect the other joints following knee arthroplasty.

## Highlights

• Joint loading and function was assessed bilaterally in unilateral knee OA.
• Gait data can be summarised using a functional classification approach.
• Gait abnormailities in knee OA and following arthroplasty are relatively symmetrical.
• Joint loading and function frequently remains abnormal following arthroplasty.
• Pre-operative function (the Cardiff Classifier) can predict post-operative function.

## 1 Introduction

Knee osteoarthritis (OA) is a common problem, and disease in one joint frequently progresses to involve both knees with time [1], [2], [3]. The second knee is a frequent source of ongoing disability after knee replacement and by 10 years approximately 40% of patients will have the other side replaced [4], [5]. There are numerous gait changes in knee OA that might be expected to impact upon the other leg, such as changes in gait speed, loading response and trunk sway [6], [7], [8], [9]. These abnormalities do not necessarily recover after total knee replacement and gait rarely returns to normal [10], [11], [12], [13].

The measurement of gait in knee OA is challenging, and principal component analysis has been used before to extract and summarise the most useful information from waveforms [14], [15], [16]. However, variables taken individually often fail to adequately describe the functional performance of gait, whereas a combination of multiple pieces of data available in a gait analysis can provide a much more comprehensive assessment [13], [16].

**Fig. 1:**
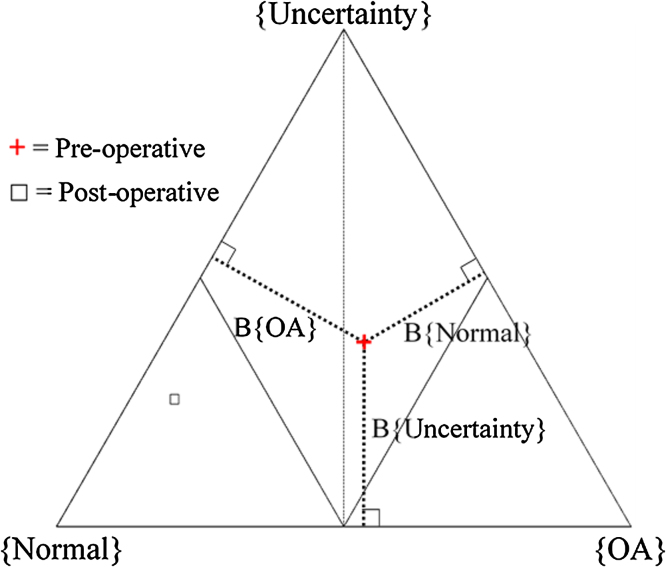
Example of a Cardiff Classifier simplex plot for a single patient demonstrating an improvement in function, with the red cross representing the pre-operative position and the black square representing the post-operative position. The position in the plot is established according to three belief values: B{OA}; B{Normal} and; B{Uncertainty}. These values are represented by the distance of the thick dotted lines in the diagram and are the shortest distances between the plotted point and the edges of the triangle. (For interpretation of the references to colour in this figure legend, the reader is referred to the web version of this article.)

In our unit, gait abnormalities have previously been described using a method based on the Dempster-Shafer theory of evidence, which provides a method for assessing mathematical probabilities that allows for uncertainty [13], [16], [17], [18]. This is particularly helpful in complex datasets such as gait data where data may be either conflicting or inconclusive. Each individual piece of data can be weighted according to its ability to contribute to the decision process, and then multiple pieces of data are mathematically combined to give 3 belief values for each subject, representing a belief in pathology, a belief in normality and a belief in uncertainty. These three belief values can be plotted on a simplex plot to give a single point for each subject summarising their gait (Fig. 1) [13], [16].

In a previous study of 20 patients who were carefully selected as having unilateral severe knee OA, abnormal moments were observed during gait in both knees and hips and elevated co-contraction was observed in both legs [19]. These patients were followed up 1 year after knee arthroplasty and provisional results in terms of joint moments and co-contraction were published in an extended conference paper (prior to completion of the full data set) [20]. However, the use of principal component analysis and classification of the data allows for a much more comprehensive analysis of gait and functional deficiencies in this group, including the relationship between objectively assessed function before and after arthroplasty. This would be expected to improve our understanding of functional deficiencies in either limb that may be targeted by rehabilitation, to establish the inter-relationship in biomechanics between the two limbs, and to better describe the changes in gait before and after knee surgery.

The aim of this paper is to review the final results of the study, to distinguish the primary features of arthritic gait in both the affected and unaffected legs relative to a normal population and to assess the objective recovery of gait function post-operatively, with the aim of defining patients who might be at risk of poor post-operative function.

## 2 Methods

### 2.1 Recruitment and patient selection

The recruitment and gait analysis process has been described in previous papers and will be summarised briefly here [19], [20]. The study was approved by the Local Research Ethics Committee and full informed consent was taken. The radiographs and clinical notes of 610 consecutive patients on the arthroplasty waiting list of two local hospitals were reviewed. The inclusion criteria were patients with medial compartment disease on the waiting list for unilateral total or uni-compartmental knee replacement. The exclusion criteria were: Any past or present history of discomfort in any other lower limb joint than the one for replacement (assessed by direct questioning, assessment of the clinical notes, and a formal examination by an orthopaedic surgeon); current lower back pain; previous surgery or trauma to the lower limbs, pelvis or spine; medical co-morbidities affecting gait; neurological disease; diabetic neuropathy; age over 85; and BMI over 40.

Twenty patients were included in the study and underwent gait analysis and electromyography. Twelve months after the joint replacement, patients invited to re-attend for a repeat assessment. WOMAC and Oxford scores were recorded at each visit.

For a control group, twenty subjects between the ages of 60 and 85 were recruited, although a younger-aged control group (age 20–60) was used for the EMG analysis, as discussed previously [19]. The control subjects all had no history of lower limb pains or disorders, no history of stroke or neurological disease and all had BMIs of less than 40.

### 2.2 Gait analysis protocol

The gait laboratory has 12 Vicon Mx2 Cameras sampled at 100 Hz and three AMTI force plates sampled at 1000 Hz. The plug-in gait marker set was used. The knee alignment device was used to define the knee centre. Subjects were requested to walk barefoot at self-selected speed. Markers were then removed and surface electrodes were placed over the palpable muscle bulk of the vastus medialis, vastus lateralis, semitendinosus and biceps femoris muscles of both legs. Electromyographic data was collected at 1000 Hz for six walking trials at self-selected speed.

External peak and mid-stance moments and moment impulses normalised to height and weight. Adduction moment impulses were calculated by integrating the whole of positive section of the curve between heel strike and toe-off [21]. Limb alignment in the coronal plane was calculated using the marker data, saving the potential radiation dose associated with x-rays [20]. Other authors have previously demonstrated good correlation between limb alignment measured using radiographic and marker-based techniques [22], [23].

Co-contraction was calculated at 100 points through stance for the medial quadriceps and hamstrings and the lateral quadriceps and hamstrings separately. The formula that was used was described previously by Lewek et al. and Ramsey et al., although values for the whole of stance were taken, rather than focusing on early stance only [19], [20], [24], [25].

### 2.3 Statistical analysis and classification

Data was processed using Excel 2007 (Microsoft, USA) and SPSS v16.01 (SPSS Inc, Illinois, USA) was used for all statistical calculations. Significance testing was performed using paired *t*-tests (for the pre-to post-operative comparisons) and independent sample *t*-tests (for the post-operative to control comparison) with alpha set at 5%. For the assessment of moments, the adduction moment impulse at the unaffected knee was defined *a priori* as the primary analysis and all other analyses were considered secondary exploratory analyses. As a result of this, multiple testing corrections were not performed, however these analyses should be considered exploratory only and should be interpreted with that in mind.

Twenty-two kinematic and kinetic waveforms from the hip, knee and ankle and 5 temporal gait parameters were extracted. The waveforms for the affected legs of the pre-operative OA subjects and the right legs of the control group were analysed using principal component analysis, according to methods described previously [14], [26]. The same analysis was then performed for the unaffected legs of OA patients and the left legs of the control group. The coefficient of variation was calculated between each data-point of the original waveform, and its new PC score. The PCs were then selected and interpreted based on the region of the original waveform where they represented at least 50% of the variation [27], [28]. The application of this criteria gave between 1 and 4 PC’s for each waveform.

The principal component scores of all of the selected waveforms for the affected legs and the 5 temporal parameters were entered into a training process for a Dempster-Shafer classifier [13], [16]. A ranking process was performed in which variables that best distinguished between OA and normal groups (using a leave one out validation method) were ranked highest. Based on previous work finding that between 15 and 20 variables were recommended for accurate classification, the highest ranking 17 variables were brought forward to be used for training the definitive classifier [28].

The same process was repeated for the data from the unaffected leg in comparison to the left legs of the controls. Two classifiers were therefore developed, one trained on a scale from normal to pathological based on data from the affected leg only and normal controls, and the other trained on a scale from normal to pathological based on data from the unaffected leg only and normal controls. The training process sets the control parameters for the variables in a single step based on the mean and standard variation of the data for that variable and is not optimised iteratively, reducing the risk of over-training. Whilst iterative optimization may be able to improve the classification accuracy further, over-training can be a risk and classification accuracy has been good in previous studies, therefore an iterative process is not used in this technique [26], [28].

Principal component scores for the post-operative waveforms were generated by multiplying standardised scores for each time point by the eigenvectors from the pre-operative training process, giving principal component scores for the post-operative data expressed on a scale defined by the pre-operative training process. These were then converted into belief values and combined as described previously, enabling the post-operative results to be plotted on the classifiers trained on the pre-operative data [16].

The plots were examined visually for patterns such as clustering of outcomes, and the pre-operative data was re-examined to determine the pre-operative factors that differentiated those patients who were likely to have either good or poor post-operative functional outcomes. As numbers for these comparisons were small, non-parametric tests were preferred therefore Mann-Whitney *U* tests were used for significance testing of differences between the identified sub-groups. It should be remembered that these analyses were performed on very low sample sizes as an exploratory analysis, and therefore multiple testing corrections or more complex analyses were not performed. These results should therefore be interpreted with some caution.

## 3 Results

Of the initial 20 OA subjects, two subjects did not have their surgery as planned, and three subjects were unable to return for reassessment after their arthroplasty due to changes in their health status or personal circumstances. Therefore 15 subjects returned for reassessment. The follow up appointment was a mean of 14.0 months post-operatively, with all but one case between 11.9 and 16.9 months (SD 1.3, range 11.9–24.7 months).

The WOMAC score fell from a mean of 48.4 (SD 12.1, range 16–65) pre-operatively to 10.5 (SD 17.5, range 0–72) post operatively, and the mean Oxford Knee Score changed from 24.2 (SD 5.7, range 12–33) pre-operatively to 40.8 (SD 8.5, range 12–48) post operatively. There were no early revisions and one re-operation, a manipulation under anaesthetic 3 months post-operatively for stiffness.

Table 1 documents temporal values and limb alignment in the OA subjects in comparison to the age-equivalent control group. Frontal plane moments at the knees and hips showed some improvement following surgery but did not return to normal (Table 2), particularly on the unaffected side. This was also true for co-contraction, although no improvement was seen in mean co-contraction on the unaffected side.

**Table 1:** Demographics, temporal parameters and limb alignment in the study population and controls. Expressed as mean (± SD).

	Pre-operative	Post-operative	Healthy Controls
Age (at pre-operative visit)	67.8 (7.2)	n/a	68.3 (5.9)
BMI	31.4 (4.0)	n/a	26.3 (3.6)
Male:Female	6:9	n/a	10:10
Gait Speed (ms^−1^)	0.97 (0.20)	1.12 (0.18)	1.33 (0.21)
Cadence (steps/min)	107 (10)	114 (8)	118 (9)

Stance percentage			
Affected limb	62.3% (2.1%)	62.0% (1.8%)	60.5% (1.6%)
Unaffected Limb	64.6% (2.8%)	62.1% (2.4%)	
Step Width (cm)	19.0 (4.2)	18.0 (4.2)	15.5 (3.4)

Limb Alignment			
Affected Limb	3.3° Varus (5.4°)	0.4° Varus (4.2°)	0.1° Valgus (2.9°)
Unaffected Limb	1.2° Valgus (2.6°)	0.4° Valgus (2.5°)

**Table 2:** Moments and co-contractions pre-and post-operatively expressed as mean (± 95% confidence interval).

	Pre-op Mean (±95%CI)	Post-op Mean (±95%CI)	Controls Mean (±95%CI)	Pre-Post change *p*-values	Post-op to control *p*-values
Hip Adduction Moments (N m/BW Ht)					
Peak					
Affected side	4.38 (0.72)	4.40 (0.51)	4.71 (0.33)	p = 0.973	p = 0.202
Unaffected side	4.70 (0.49)	4.87 (0.46)		p = 0.383	p = 0.332
Mid-stance					
Affected side	3.82 (0.54)	3.43 (0.45)	2.70 (0.29)	p = 0.155	p = 0.010*
Unaffected side	3.66 (0.52)	3.46 (0.35)		p = 0.203	p = 0.005**

Knee Adduction Moments (N m/BW Ht)					
Peak					
** **Affected side	3.11 (0.40)	2.52 (0.31)	3.09 (0.42)	p = 0.021*	p = 0.037*
Unaffected side	2.78 (0.36)	2.88 (0.44)		p = 0.654	p = 0.464
Mid-stance					
Affected side	2.25 (0.43)	1.58 (0.34)	0.94 (0.25)	p = 0.003**	p = 0.004**
Unaffected side	1.70 (0.35)	1.55 (0.30)		p = 0.352	p = 0.008**

Adduction Moment Impulse (N m s/BW Ht)					
Hip					
Affected side	2.05 (0.33)	1.84 (0.20)	1.76 (0.13)	p = 0.164	p = 0.646
Unaffected side	2.34 (0.29)	1.98 (0.16)		p = 0.003**	p = 0.045*
Knee					
Affected side	1.32 (0.19)	0.89 (0.17)	0.84 (0.12)	p < 0.001**	p = 0.418
Unaffected side	1.09 (0.18)	0.99 (0.16)		p = 0.132	p = 0.164

Medial Co-contraction Index					
Affected side	0.27 (0.03)	0.22 (0.03)	0.14 (0.02)	p = 0.018*	p < 0.001**
Unaffected side	0.18 (0.03)	0.18 (0.04)		p = 0.74	p = 0.17

Lateral Co-contraction Index					
Affected side	0.28 (0.04)	0.24 (0.04)	0.15 (0.03)	p = 0.071	p = 0.003**
Unaffected side	0.22 (0.05)	0.21 (0.04)		p = 0.74	p = 0.016*

* Significant (p < 0.05).

** Highly significant (p < 0.01).

**Fig. 2:**
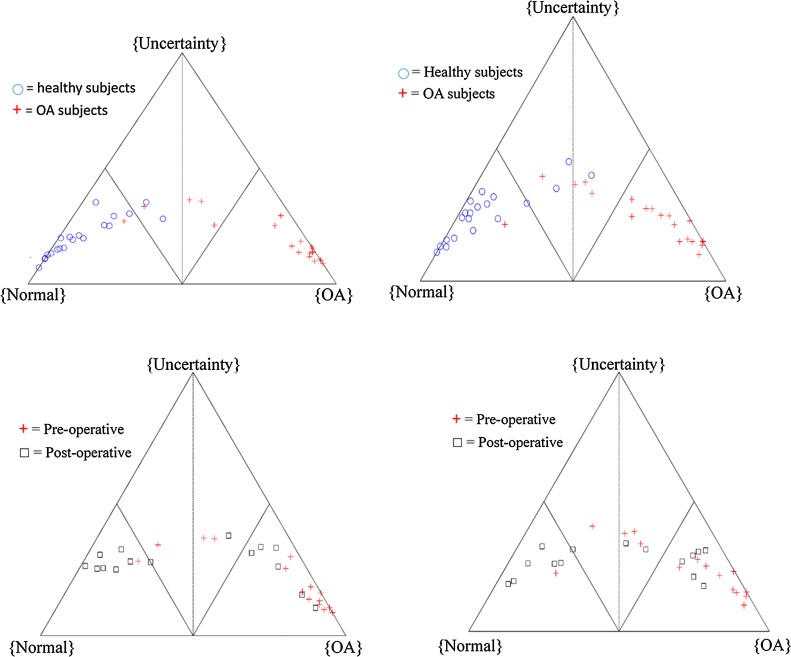
Cardiff Classifier results: a) and b) are from the training process (affected leg and unaffected leg respectively), showing the clear separation between the healthy (blue circles) and the OA subjects (red crosses). c) and d) demonstrate the change from pre-operative (red crosses) to post-operative (black squares) results (affected and unaffected legs respectively). (For interpretation of the references to colour in this figure legend, the reader is referred to the web version of this article.)

The highest ranked variable was hip power in both the affected and unaffected legs. The rankings and classification accuracies are included in the Supplementary material, as are charts comparing hip, knee and ankle powers (which were all highly ranked) between the groups. There were 2 mis-classifications at the affected leg and 3 mis-classifications at the unaffected leg (Fig. 2a and b). The classification accuracy for the pre-operative classifier was 95% for the affected leg and 92.5% for the unaffected leg.

In Fig. 2c) and d), the post-operative data for the 15 OA subjects is plotted against the pre-operative data for the same 15 subjects. A point close to the B[OA] vertex would represent a high belief in OA function, a point close to B[NL] would represent a high belief in normal function, and a high B[Θ] would represent a high level of uncertainty in the data. Uncertainty can be the result of conflicts in the data or individual variables which do not differentiate well between categories [16].

At the affected knee, two groups of post-operative functional outcome were seen, with eight subjects having moved well over to the normal half of the classifier and seven subjects remaining on the OA side of the classifier (Fig. 2c). At the unaffected knee there were also two clusters of functional outcome seen, with seven patients having relatively normal function, whereas eight patients demonstrated a pattern that was more typical of OA (Fig. 2d). One subject changed to the normal half based on the affected leg but did not cross the midline in the unaffected leg.

When the pre-operative results for these clusters were examined, the clusters did not differ significantly in terms of BMI (p = 0.456), pre-op Oxford score (p = 0.259) or pre-op WOMAC score (p = 0.902) but there was a non-significant trend towards a difference in age (mean 64.9 for good response group and 71.9 for poor response group, p = 0.053) and significant differences in pre-operative belief in OA (mean 0.550 for good response group and 0.841 for poor response group, p = 0.017), belief in normal function (mean 0.173 for good response group and 0.022 for poor response group, p = 0.026) and belief in uncertainty (mean 0.278 for good response group and 0.137 for poor response group, p = 0.017).

**Fig. 3:**
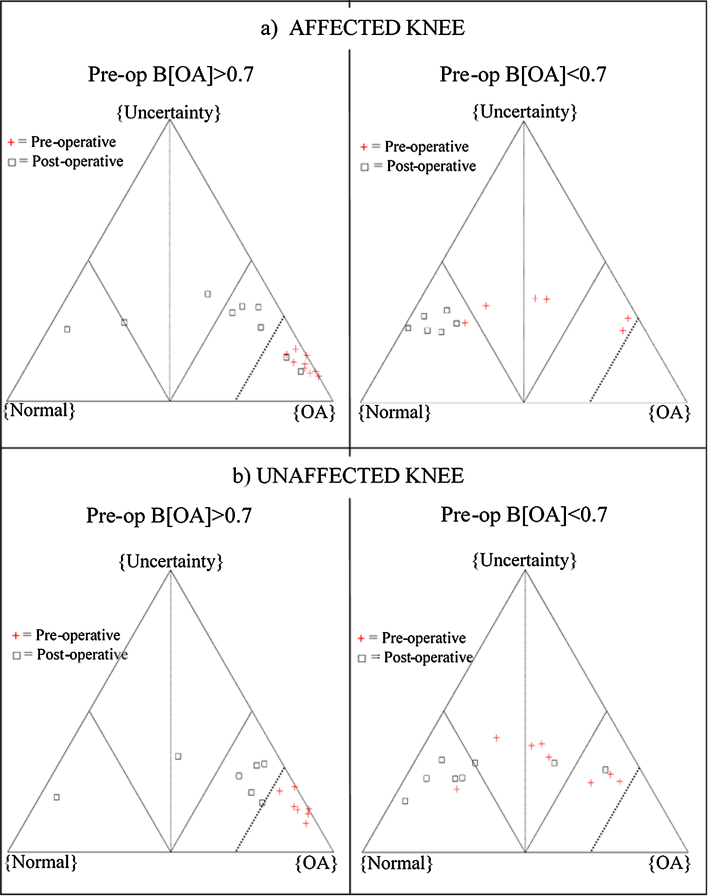
The use of thresholds to define post-operative results according to pre-operative results at a) the affected leg and b) the unaffected leg. Pre-operative (red cross) and post-operative (black square) objectively assessed function is plotted for those with ‘poor’ pre-op function (defined as B[OA] > 0.7) on the left and ‘good’ pre-op function (B[OA] < 0.7) on the right. The thick dashed line represents B[OA] = 0.7. (For interpretation of the references to colour in this figure legend, the reader is referred to the web version of this article.)

A pre-operative belief in OA of 0.7 was identified as the threshold above which poor pre-operative function could be expected in both legs. At the affected knee, a cut off of pre-operative function of B[OA] < 0.7 determined whether the post-operative score was in the ‘normal’ or ‘osteoarthritic’ half of the classifier 13 out of 15 times (Fig. 3a). Using data from the unaffected limb, this was only slightly less effective, as a cut-off of pre-operative function of B[OA] < 0.7 determined whether the post-operative score was in the ‘normal’ or ‘osteoarthritic’ half of the classifier 12 out of 15 times (Fig. 3b).

## 4 Discussion

Knee OA is classically referred to as an ‘asymmetric arthropathy’ however gait changes in knee OA show high levels of symmetry, even when only one knee is affected.

After knee replacement, moments at the affected knee fell significantly whilst the change was variable at the unaffected leg, with only a moderate change overall. It would be prudent to advise patients that ‘protecting the other knee’ is not a valid reason for progressing with knee replacement based on this data. This was a highly selected cohort of patients without contralateral knee or hip pain and despite the successful treatment of their painful knee, gait abnormalities persist.

Gait changes in knee OA should not be simply considered ‘antalgic’, implying that one leg compensates for the other, but instead are characterised by bilateral reductions in peak joint powers, changes in the ground reaction vector, reduced gait speed, and abnormal muscle co-contractions. These changes are sufficiently consistent that the gait patterns can be distinguished mathematically using data from either the affected or unaffected knee, without any input data other than gait waveforms and temporal data. Post-operatively, function improves in the majority, but the magnitude of recovery varies significantly between individuals and it often does not normalise.

Recovery of objectively measured function was remarkably predicable in this study and strongly dependent upon pre-operative function, with a threshold set in the pre-operative data able to correctly predict whether subjects would return to the normal half of the classifier post-operatively with 87% accuracy using pre-operative data from the affected leg and with 80% accuracy using data from the unaffected leg. This data may be valuable in developing predictive models in the future, allowing clinicians to set better thresholds for patient-specific treatment decisions. It also raises the importance of addressing biomechanical abnormalities pre-operatively with focused rehabilitation, as better function before surgery would be likely to improve function after surgery.

The study has a number of weaknesses which should be acknowledged. The cohort was a highly selected group of patients with single joint disease. This had the advantage of ensuring that abnormal biomechanics in the other knee could not be due to either deformity or pain, as the presence of either (even small amounts of pain, ache or ‘niggle’) were strict exclusion criteria to the study and this was confirmed by formal clinical examination. As such, a radiograph of the other knee was not indicated, avoiding the additional radiation dose, as a normally aligned knee without pain should have no effect on gait. However, further work is required to determine whether these findings can be generalised to the broader knee OA population who often have some degree of pain elsewhere.

The use of a strictly defined study population allowed for a detailed analysis of the biomechanics of both the affected and the unaffected limb, but it also resulted in a study with a small sample size. This should be considered carefully when analysing these results, especially when comparing the two clusters of outcomes where numbers for the analysis were very low. The analysis should be treated as exploratory only and multiple testing corrections were not performed as they may be considered too conservative. A study such as this should be considered hypothesis generating and should stimulate future work to confirm or refute the findings.

The recovery of function is variable between patients and this is demonstrated in this study, where two clusters of functional outcome were seen. Whilst the midline of the classifier was used to define the two groups, this is an arbitrary marker and different thresholds may be more appropriate to different patient cohorts. Also, the development of a threshold in this study was performed *post hoc* and future studies are required to test this observation on new patients using thresholds that have been identified *a priori*. The pre-operative data was also used to train the classifier, and future analysis may be better performed with a distinct training set. Further analysis on large, less selected, more representative cohorts is planned for the future.

## 5 Conclusion

Gait is consistently abnormal in unilateral severe knee OA, and the changes are observed in both the affected and unaffected legs. Post-operative recovery in gait is highly variable, but the resolution of normal gait can be predicted pre-operatively in the majority of individuals, raising the possibility of developing thresholds for surgery or pre-operative training aimed at optimising outcome in the future.

## Conflicts of interest

There are no conflicts of interest to declare.
