# Supplementary material for: Abnormal loading and functional deficits are present in both limbs before and after unilateral knee arthroplasty
Source: Gait Posture. 2017 Jun;55:109–15. doi: 10.1016/j.gaitpost.2017.04.008 (PMC5450932; doi:10.1016/j.gaitpost.2017.04.008)

Supplementary table 1. Ranking order and classification accuracy of the different variables used in the classifiers. Each variable was represented by its highest ranked principal component, except for temporal measures where the raw data was included. Classification accuracies are given for each individual variable and are not cumulative (GRF-V = Ground reaction force vertical component, GRF-AP = Ground reaction force antero-posterior component).

| **Affected Knee** | | |  | **Unaffected knee** | | |
| --- | --- | --- | --- | --- | --- | --- |
| **Rank** | **Variable** | **Classification accuracy** |  | **Rank** | **Variable** | **Classification accuracy** |
| 1 | Hip Power | 92.5% |  | 1 | Hip Power | 87.5% |
| 2 | Knee Power | 87.5% |  | 2 | Sagittal Knee Angle | 85% |
| 3 | GRF-V | 85% |  | 3 | Knee Power | 82.5% |
| 4 | Sagittal Knee Moment | 85% |  | 4 | Double Support Time | 80% |
| 5 | Sagittal Hip Moment | 85% |  | 5 | Gait Speed | 80% |
| 6 | Double Support Time | 82.5% |  | 6 | Sagittal Knee Moment | 80% |
| 7 | GRF-AP | 82.5% |  | 7 | Step Time | 77.5% |
| 8 | Gait Speed | 80% |  | 8 | Sagittal Hip Moment | 77.5% |
| 9 | Saggital Ankle Dorsi/Plantar | 80% |  | 9 | GRF-V | 77.5% |
| 10 | Coronal Hip Moment | 80% |  | 10 | Saggital ankle | 75% |
| 11 | Ankle Power | 77.5% |  | 11 | Axial Hip | 72.5% |
| 12 | Step Length | 75% |  | 12 | Ankle Power | 72.5% |
| 13 | Sagittal Ankle Moment | 75% |  | 13 | Sagittal hip | 70% |
| 14 | Coronal Knee Moment | 72.5% |  | 14 | Step width | 65% |
| 15 | Step Width | 70% |  | 15 | GRF-AP | 65% |
| 16 | Coronal Hip Angle  (Ab/Ad-duction) | 70% |  | 16 | Coronal Knee Moment | 65% |
| 17 | Sagittal Pelvis | 62.5% |  | 17 | Sagittal Ankle Moment | 65% |

Supplementary figure 1. Mean powers for hip knee and ankle in both legs, at; a) the hip b) the knee and c) the ankle (dotted line = healthy subjects, continuous line = OA pre-op, broken line = post-operative).


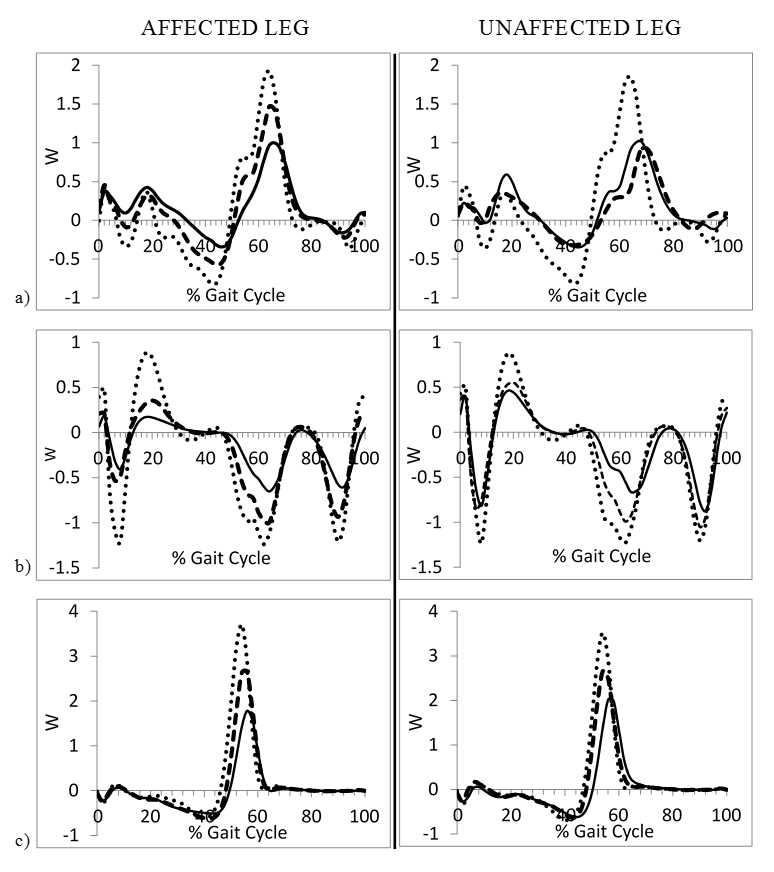

Supplement: Supplementary file 1 [file mmc1.docx]
